# The impact of personalized clinical decision support on primary care patients’ views of cancer prevention and screening: a cross-sectional survey

**DOI:** 10.1186/s12913-021-06551-9

**Published:** 2021-06-21

**Authors:** Daniel M. Saman, Ella A. Chrenka, Melissa L. Harry, Clayton I. Allen, Laura A. Freitag, Stephen E. Asche, Anjali R. Truitt, Heidi L. Ekstrom, Patrick J. O’Connor, JoAnn M. Sperl-Hillen, Jeanette Y. Ziegenfuss, Thomas E. Elliott

**Affiliations:** 1 Nicklaus Children’s Health System, 3601 NW 107th Ave, Doral, FL 33178 USA; 2 Essentia Institute of Rural Health, 502 E. Second Street, Duluth, MN 55805 USA; 3 HealthPartners Institute, 3311 E. Old Shakopee Road, Bloomington, MN 55425 USA

**Keywords:** Cancer prevention, Cancer screening, Clinical decision support, Decision aid, Electronic health record, Patient survey

## Abstract

**Background:**

Few studies have assessed the impact of clinical decision support (CDS), with or without shared decision-making tools (SDMTs), on patients’ perceptions of cancer screening or prevention in primary care settings. This cross-sectional survey was conducted to understand primary care patient’s perceptions on cancer screening or prevention.

**Methods:**

We mailed surveys (10/2018–1/2019) to 749 patients aged 18 to 75 years within 15 days after an index clinical encounter at 36 primary care clinics participating in a clinic-randomized control trial of a CDS system for cancer prevention. All patients were overdue for cancer screening or human papillomavirus vaccination. The survey compared respondents’ answers by study arm: usual care; CDS; or CDS + SDMT.

**Results:**

Of 387 respondents (52% response rate), 73% reported having enough time to discuss cancer prevention options with their primary care provider (PCP), 64% reported their PCP explained the benefits of the cancer screening choice very well, and 32% of obese patients reported discussing weight management, with two-thirds reporting selecting a weight management intervention. Usual care respondents were significantly more likely to decide on colorectal cancer screening than CDS respondents (*p* < 0.01), and on tobacco cessation than CDS + SDMT respondents (*p* = 0.02) and both CDS and CDS + SDMT respondents (*p* < 0.001).

**Conclusions:**

Most patients reported discussing cancer prevention needs with PCPs, with few significant differences between the three study arms in patient-reported cancer prevention care. Upcoming research will assess differences in screening and vaccination rates between study arms during the post-intervention follow-up period.

**Trial registration:**

clinicaltrials.gov, NCT02986230, December 6, 2016.

**Supplementary Information:**

The online version contains supplementary material available at 10.1186/s12913-021-06551-9.

## Background

In 2017, 21% of all U.S. deaths were caused by cancers [1]. While overall cancer death rates have dropped [2, 3], prevalence and lifetime risk of breast, cervical, colorectal, and lung cancer remain substantial [2], and screening rates are still insufficient. Both breast and cervical cancer screening rates have declined generally in recent years (2000–2015), while colorectal cancer screening rates have increased (38 to 63%) during the same time period, and the low rate of lung cancer screening has stayed the same at over 3% [4].

There is strong evidence that early diagnosis of breast, cervical, lung (for current or past smokers who qualify), and colorectal cancer may help lower mortality rates and lessen cancer morbidity, even when adjusting for lead-time bias [2, 5]. For example, low-dose Computed Tomography (LDCT) scans for people with a 30 or more pack-year smoking history has been shown to lower lung cancer mortality rates by 20% [6]. Unfortunately, often times patients forgo LDCT screening, most notably because they may not understand the information given to them, they do not think the test is worth the time, there are barriers that prevent them from getting the test done, they are concerned about getting a false-positive, or they simply do not want to know [7]. When it comes to having a colonoscopy procedure, around 75% of patients cite bowel preparation and fear as the main reasons for not wanting the procedure [8]. Mammograms, a mainstay for breast cancer detection, have both benefits and risks, the latter of which include false positives, false negatives, radiation, and unnecessary surgery [9]. Cervical cancer may have the most benign screening method, the Pap smear [10], as well as an effective HPV vaccine that has struggled to achieve widespread uptake [11–13].

Tailored clinical decision support (CDS) that includes cancer screening and prevention recommendations to both patients and primary care providers (PCPs) at primary care encounters may increase screening rates and improve prevention efforts [14, 15]. For example, there is some evidence that CDS, in the form of simple prompts or reminders, can lead to improved mammogram rates and higher rates of use for certain other cancer prevention services [14, 16–18]. Yet a prior systematic review showed that observed upticks in preventive service related to prompts and reminders were relatively small [16]. While a more recent systematic review suggests some CDS designs and workflows facilitate use more than others [17], we still have an incomplete understanding of whether CDS that goes beyond simple prompts and reminders may further improve delivery of cancer preventive care.

We also have an incomplete understanding of whether shared decision making between patients and PCPs leads to improved cancer preventive care. Shared decision making involves PCPs presenting patients with cancer prevention and screening options, risk, and benefits, including when using decision aids, then assisting patients with making a decision that fits patients’ personal preferences [19, 20]. Research shows that shared decision making between patients and PCPs is effective in the decision making process [18]. However, delivery of personalized decision aids in clinical practice is challenging. It is feasible to use CDS systems as a vector to deliver visual decision aids, including those referred to as shared decision-making tools (SDMT), to patients during an office-based clinical encounter.

To address these current gaps in knowledge on the impact of CDS and SDMT decision aids on cancer prevention care, we designed a CDS that can print tailored materials for patients and PCPs when patients are noted in the electronic health record (EHR) as in need of primary and secondary cancer prevention and screening. We undertook a three-arm, clinic-randomized control trial (RCT) to assess whether CDS alone, or CDS plus SDMT, can improve cancer preventive care compared to usual care in the control arm. The objective of the present cross-sectional survey was to assess the impact of cancer prevention CDS, with or without SDMT, on patients’ self-reported experiences with and perceptions of the cancer prevention care they received at an initial (study-design index) primary care clinic visit.

## Methods

### Study population

This study was conducted at a large, primarily rural integrated healthcare system with 69 clinics and 13 hospitals in three upper Midwestern states (Minnesota, North Dakota, and Wisconsin). As we noted in a prior paper, “preliminary data from 2012 to 2014 among eligible Essentia Health patients aged 11–80 with two or more primary care visits within 36 months showed about two-thirds are up to date on colorectal cancer screening, two-thirds up to date on breast cancer screening, 54% up to date on cervical cancer screening, and 5% of males aged 11–26 and 20% of females are up to date on HPV vaccination” (p. 2) [15]. This suggests significant gaps in cancer screening and HPV vaccination within the study population. The study sample size was determined based on the minimum needed to ascertain bivariate differences between intervention and usual care.

Patients eligible for the cross-sectional cohort survey met the following criteria: age 18 to 75; visited a study clinic over a 4-month period; due or overdue for at least one primary cancer prevention (tobacco cessation, referral for tobacco cessation counseling, prescription of smoking cessation medication, HPV vaccination) or secondary cancer prevention (screening for breast, colorectal, cervical, or lung cancer); not receiving hospice care; no active cancer (except non-melanoma skin cancer) in the last year; and not pregnant. Specific prevention and screening-related eligibility included: breast cancer – average risk women ages 50–75 or above average risk (BCRAT > 2% or lifetime > 16.8%) women ages 35–49 [21]; cervical cancer women ages 21–65 [22]; colorectal cancer screening – high risk adults ages 18–75, non-high risk adults ages 50–75 or ages 40–75 with a documented family history or polyps [23]; HPV vaccine: ages 18–26 [24]; lung cancer screening: adults ages 55–75 with a 30-year pack habit [25]; current tobacco use ages 18–75; and BMI > 25 ages 18–75. This study was reviewed in advance, approved, and monitored by the Essentia Health Institutional Review Board. All methods were performed in accordance with relevant institutional and federal guidelines and regulations. The Essentia Health Institutional Review Board waived the requirement of documentation of informed consent for this survey; therefore, written informed consent was not required or obtained for survey participants. Both the mailed invitation letter and the telephone script stated that: “completing this survey lets us know that you consent to participate in this research study.” All respondents to this survey were age 18 and over.

### Intervention

The survey was conducted within a larger RCT on the use of a CDS intervention within 36 primary care clinics randomized across three study arms: (a) usual care (control), (b) cancer prevention CDS alone (CDS), and (c) cancer prevention CDS enhanced with SDMT (CDS + SDMT) [15, 19, 26]. In both intervention arms, a point-of-care and EHR-linked CDS, known as Priorities Wizard [27], used several sets of web-based algorithms to identify evidence-based cancer prevention care options that address unmet breast, cervical, lung, or colorectal cancer prevention needs. PCPs and their patients in the CDS + SDMT intervention arm, in addition to cancer prevention CDS, also had access to short 2–4 page SDMT developed by the study team for patients to more easily understand their personal cancer risk, as well as the risk and benefits of each cancer screening [26]. The study team modified existing evidence based SDMT based on current literature, expert opinion, and pilot testing [26]. Rooming staff received an EHR alert for eligible patients instructing them to print the CDS or CDS + SDMT materials (depending on clinic-randomized intervention arm), giving an abbreviated version of the CDS printout (and any SDMT materials) to the patient and placing a more detailed version for the PCP on the exam room door [20].

In the usual care arm of the study, clinics and their PCPs had no access to CDS or CDS + SDMT. Participants included in the usual care study arm would have met the criteria for the CDS if they visited an intervention clinic. We recently published a paper outlining the design of the RCT [28]. Results of the RCT will be published separately. The CDS supports Wagner’s model for improving chronic illness care by: providing patients with cancer prevention and screening information tailored to their needs; incorporating members of the care team (rooming staff, PCPs); and implementing targeted evidence-based guidelines and support within the EHR [29].

### Survey instrument

The patient questionnaire included questions on patient demographics, whether or not a cancer prevention and screening option was discussed and what action was taken, discussions with clinicians and care teams [30], whether their clinician allocated sufficient time to discuss cancer prevention options, and other questions regarding cancer prevention developed by the study team (Additional file 1). The survey also included the low literacy, 10-item English version of the Decisional Conflict Scale (DCS) that is scored on a 0 to 100 scale [31]. Demographic information obtained included sex, age, education, race, household composition, employment status, and household income.

### Data collection

Between October 2018 and January 2019, a randomly selected sample of patients in each study arm was mailed a paper survey within 15 days of their study-defined primary care index visit. As this was an exploratory cross-sectional study, we determined a-priori that a sample size around *n* = 100 in each study arm would be appropriate for understanding differences by arms that our budget could afford. The mailed survey included an invitation letter stating that survey completion was optional, a paper survey, a $2 bill, and a postage-paid return envelope. Patients were asked to complete the survey either by mail or telephone. Completed survey data were posted to an online reporting tool (REDCap) [32] for review by the study team. After 3 weeks, non-respondents received a phone call (with up to seven total phone call attempts) to complete the survey either by mail or phone. Respondents who completed the survey over the phone were asked the same questions included in the mailed survey. Patients who completed the survey received a $10 gift card. Phone contact attempts continued until 100 patients from each of the three arms completed the survey. All survey data collection was coordinated and completed by the Center for Evaluation and Survey Research at HealthPartners Institute.

### Data analysis

Prior to data analysis, we reviewed all response data for out of range values and restricted denominators for those eligible for certain sections (e.g., removed responses for screenings for which respondents were not eligible). Only available data were used for each survey item. Responses to demographic and general health items were summarized for the whole sample, and for each study arm. Survey responses were aggregated and summarized by group.

For each of the seven cancer prevention services, the proportion of eligible patients that recalled having a discussion relevant to screening or treatment options at their index primary care visit was calculated. Of those eligible who recalled a discussion, the proportion who decided to get screening or other relevant treatment was presented as well. For all single items, chi-square or Fisher’s exact test (as appropriate) were used to compare the distribution of responses in the usual care group to the distribution of responses and a combined group of the two intervention groups (CDS, CDS + SDMT) in 2 × 2 tables (comparisons between individual arms were also made, with selected significant results reported). The Freeman-Halton test (also referred to as Fisher’s exact test or Fisher-Freeman-Halton test) that extends Fisher’s exact test to tables larger that 2 × 2 was used for all *R*x*C* tables with cell counts < 5. DCS scale scores were compared between study arms using *t*-tests and ANOVA. No adjustments were made for multiple comparisons. Comparisons with two-tailed *p*-values < 0.05 were considered statistically significant. All analyses were performed using SAS v. 9.4 in 2020.

## Results

We mailed surveys to 749 patients; six were returned with an incorrect address and one respondent self-reported as not eligible. Of the 742 eligible patients remaining, 387 (52%, response rate) completed the survey, with 373 completing by mail and 14 by phone. Of these responders, 383 were eligible for at least one of the screenings or interventions and were used as our analytic sample. In total, 18% (*n* = 68) were eligible for breast cancer screening, 22% (*n* = 83) for cervical cancer screening, 45% (*n* = 171) for colorectal cancer screening, 17% (*n* = 64) for lung cancer screening, 34% (*n* = 132) for smoking cessation intervention, and 74% for BMI (*n* = 286). The average responder was eligible for two screenings or interventions (*M* = 2.1, *SD* = 0.9, range 1–6), and 299 (78%) were eligible for 2 or more. Table 1 shows characteristics of survey respondents by study arm. Seventy percent of respondents were female and 96% were White.

**Table 1:** Respondent demographics

			Intervention Arms	
Survey Items	All Arms	UC	CDS	CDS + SDMT
Gender	(*N* = 383)	(*N* = 119)	(*N* = 129)	(*N* = 135)
Female	268 (70)	91 (77)	86 (67)	91 (67)
Male	115 (30)	28 (24)	43 (33)	44 (33)
Age Range in Years	(*N* = 383)	(*N* = 119)	(*N* = 129)	(*N* = 135)
18–26	16 (4)	6 (5)	6 (5)	4 (3)
27–39	44 (12)	19 (16)	9 (7)	16 (12)
40–49	27 (7)	10 (8)	9 (7)	8 (6)
50–59	111 (29)	37 (31)	37 (29)	37 (27)
60–69	134 (35)	35 (29)	49 (38)	50 (37)
≥ 70	51 (13)	12 (31)	19 (34)	20 (36)
Highest Grade or Level of School	(*N* = 371)	(*N* = 112)	(*N* = 128)	(*N* = 131)
8th grade or less	3 (1)	1 (1)	1 (1)	1 (1)
Some high school	20 (5)	6 (5)	7 (6)	7 (5)
High school graduate or GED	98 (26)	32 (29)	31 (24)	35 (27)
Some college or 2-year degree	167 (45)	54 (48)	57 (45)	56 (43)
4-year college graduate	41 (11)	6 (5)	17 (13)	18 (14)
More than 4-year college degree	42 (11)	13 (12)	15 (12)	14 (11)
Hispanic or Latino	(*N* = 374)	(*N* = 116)	(*N* = 125)	(*N* = 133)
Yes	4 (1)	3 (3)	1 (1)	0 (0)
Race/Ethnicity	(*N* = 382)	(*N* = 120)	(*N* = 129)	(*N* = 138)
American Indian/Alaska Native	3 (1)	1 (1)	1 (1)	1 (1)
Asian	2 (1)	0 (0)	1 (1)	1 (1)
Black or African American	1 (< 1)	1 (1)	0 (0)	0 (0)
White	368 (96)	114 (95)	121 (94)	133 (96)
Multiple race codes	8 (2)	4 (3)	6 (5)	3 (2)
Current Employment Status	(*N* = 374)	(*N* = 116)	(*N* = 125)	(*N* = 133)
Employed for wages	166 (44)	54 (47)	52 (42)	60 (45)
Self-Employed	19 (5)	2 (2)	11 (9)	6 (5)
Out of work for > 1 year	4 (1)	1 (1)	1 (1)	2 (2)
Out of work for < 1 year	1 (< 1)	0 (0)	0 (0)	1 (1)
Homemaker	7 (2)	3 (3)	1 (1)	3 (2)
Student	6 (2)	4 (4)	1 (1)	1 (1)
Retired	123 (33)	32 (28)	45 (36)	46 (35)
Unable to work	16 (4)	4 (4)	7 (6)	5 (4)
On disability/leave of absence	32 (9)	16 (14)	7 (6)	9 (7)
Total Household Income Last Year	(*N* = 351)	(*N* = 112)	(*N* = 113)	(*N* = 126)
$0 - $25,999	121 (35)	51 (46)	37 (38)	33 (26)
$26,000 - $51,999	113 (32)	34 (30)	37 (33)	42 (33)
$52,000 - $74,999	61 (17)	15 (13)	18 (16)	28 (22)
More than $75,000	56 (16)	12 (11)	21 (19)	23 (18)

Notes. Data are n (%). Count data are shown. Percentages are rounded to the nearest percentage point. Percentages may not add up to 100% due to rounding. *CDS* Clinical decision support, *SDMT* Shared decision making tools, *UC* Usual care

### Patient and PCP cancer prevention and screening discussions and decisions made

No significant differences were found between study arms in patient self-report of having enough time to discuss cancer prevention options, or how well PCPs explained the risks and benefits of cancer prevention options (Table 2). While 73% of respondents overall reported having enough time to discuss cancer prevention options with their PCP, 12% reported that cancer risks were not explained well at all.

**Table 2:** Respondent perceptions of cancer prevention and screening discussions with their primary care providers

			Intervention Arms		
Survey Items	All Arms	UC	CDS	CDS + SDMT	*p*
Did you have enough time to discuss cancer prevention options (breast cancer, colorectal cancer, lung cancer, cervical cancer, HPV vaccine, quitting tobacco, weight management) with your provider?					
	(*N* = 286)	(*N* = 92)	(*N* = 102)	(*N* = 92)	0.40
Yes	208 (73)	64 (69)	75 (73)	69 (75)	
No	78 (27)	28 (31)	27 (27)	23 (25)	
How well did your provider explain the risks of the choices available to you?					
	(*N* = 286)	(*N* = 92)	(*N* = 102)	(*N* = 92)	0.13
Very well	187 (65)	56 (61)	70 (69)	61 (66)	
Somewhat well	64 (22)	27 (29)	20 (20)	17 (19)	
Not at all well	35 (12)	9 (10)	12 (12)	14 (15)	
How well did your provider explain the benefits of the choice available to you?					
	(*N* = 283)	(*N* = 91)	(*N* = 101)	(*N* = 91)	0.18
Very well	182 (64)	56 (62)	69 (68)	57 (63)	
Somewhat well	70 (25)	28 (31)	20 (20)	22 (24)	
Not at all well	31 (11)	7 (8)	12 (12)	12 (13)	

Notes. Data are n (%). Count data are shown. Percentages are rounded to the nearest percentage point. Percentages may not add up to 100% due to rounding. Boldface indicates statistical significance (*p* < 0.05) using chi-square test of association (df = 2) or the Freeman-Halton test where noted. Comparisons are UC to CDS and CDS + SDMT intervention arms combined. *CDS* Clinical decision support, *SDMT* Shared decision making tools, *UC* Usual care

Overall, 97% of respondents reported their PCP usually or always explained things in a way that was easy to understand, and 80% reported their care team either always or usually talked with them about specific things to prevent illness (Table 3). However, we found no significant differences between study arms.

**Table 3:** Respondent perceptions of primary care provider explanations and care team prevention discussions

			Intervention Arms		
Survey Items	All Arms	UC	CDS	CDS + SDMT	*p*
At your last primary care appointment …					
How often did your provider explain things in a way that was easy to understand?					
	(*N* = 382)	(*N* = 118)	(*N* = 129)	(*N* = 135)	0.59
Always	278 (73)	83 (70)	101 (78)	94 (70)	
Usually	91 (24)	30 (25)	27 (21)	34 (25)	
Sometimes	11 (3)	4 (3)	1 (1)	6 (4)	
Rarely	1 (< 1)	1 (1)	0 (0)	0 (0)	
Never	1 (< 1)	0 (0)	0 (0)	1 (1)	
How often did the care team talk with you about specific things you could do to prevent illness?					
	(*N* = 378)	(*N* = 119)	(*N* = 127)	(*N* = 132)	0.61
Always	154 (41)	44 (37)	60 (47)	50 (38)	
Usually	146 (39)	50 (42)	43 (34)	53 (40)	
Sometimes	56 (15)	19 (16)	15 (12)	22 (17)	
Rarely	18 (5)	6 (5)	8 (6)	4 (3)	
Never	4 (1)	0 (0)	1 (1)	3 (2)	

Notes. CAHPS Clinician & Group Surveys, 2012. Data are n (%). Count data are shown. Percentages are rounded to the nearest percentage point. Percentages may not add up to 100% due to rounding. Freeman-Halton test. Comparisons are UC to CDS and CDS + SDMT intervention arms combined. *CDS* Clinical decision support, *SDMT* Shared decision-making tools, *UC* Usual care

Regarding prevention and screening discussions between patients and PCPs and decisions made, there were no significant differences between usual care and combined intervention arms (Table 4). However, when comparing arms separately, the CDS intervention arm respondents had higher rates of reporting discussing breast cancer prevention or screening than those in the CDS + SDMT intervention arm (Fisher’s Exact, *p* = 0.03, not shown in Table 4). Furthermore, CDS intervention respondents also had higher rates of reporting discussing tobacco cessation than CDS + SDMT intervention arm respondents (Fisher’s Exact, *p* = 0.01, not shown in Table 4).

**Table 4:** Most recent primary care appointment discussions and decisions made

			Intervention Arms		
Survey Items	All (*N* = 330)	UC (*N* = 112)	CDS (*N* = 112)	CDS + SDMT (*N* = 106)	*p*
At your last primary care appointment did you discuss					
Eligible for breast cancer screening	(*N* = 62)	(*N* = 18)	(*N* = 20)	(*N* = 24)	
Yes, breast cancer discussed	42 (68)	12 (67)	17 (85)	13 (54)	0.99
Decided a screening option^a^	36 (62)	7 (58)	12 (71)	7 (54)	0.99
Decided not to get screened	16 (38)	5 (42)	5 (29)	6 (46)	
Eligible for colorectal cancer screening	(*N* = 149)	(*N* = 44)	(*N* = 52)	(*N* = 53)	
Yes, colorectal cancer discussed^b^	98 (66)	30 (68)	32 (62)	36 (68)	0.85
Decided a screening option	57 (58)	19 (63)	12 (38)	26 (72)	0.51
Decided not to get screened	41 (42)	11 (37)	20 (63)	10 (28)	
Eligible for lung cancer screening	(*N* = 51)	(*N* = 14)	(*N* = 18)	(*N* = 19)	
Yes, lung cancer discussed	14 (27)	6 (43)	3 (17)	5 (26)	0.17
Decided to get CT chest scan	5 (36)	3 (50)	0 (0)	2 (40)	0.58
Decided not to get screened	9 (64)	3 (50)	3 (100)	3 (60)	
Eligible for cervical cancer screening	(*N* = 71)	(*N* = 23)	(*N* = 18)	(*N* = 30)	
Yes, cervical cancer discussed	20 (28)	8 (35)	6 (33)	6 (20)	0.41
Decided to get a Pap smear	16 (80)	6 (75)	6 (100)	4 (67)	0.99
Decided not to get screened	4 (20)	2 (25)	0 (0)	2 (33)	
Eligible for HPV vaccination	(*N* = 9)	(*N* = 4)	(*N* = 3)	(*N* = 2)	
Yes, HPV vaccination discussed	4 (44)	1 (25)	2 (67)	1 (50)	0.52
Decided to get vaccinated^d^	1 (25)	0 (0)	1 (50)	0 (0)	0.99
Decided not to get vaccinated	3 (75)	1 (100)	1 (50)	1 (100)	
Eligible for tobacco cessation	(*N* = 127)	(*N* = 46)	(*N* = 38)	(*N* = 43)	
Yes, tobacco cessation discussed	81 (64)	30 (65)	31 (82)	20 (47)	0.85
Decided a cessation option^e^	34 (42)	18 (60)	11 (35)	5 (25)^f^	**< 0.001**
Decided to do nothing	47 (58)	12 (40)	20 (65)	15 (75)	
Eligible for weight management	(*N* = 223)	(*N* = 66)	(*N* = 73)	(*N* = 84)	
Yes, weight management discussed	71 (32)	25 (38)	21 (29)	25 (30)	0.21
Decided a management option^g^	44 (62)	17 (68)	11 (52)	16 (64)	0.61
Decided to do nothing	27 (38)	8 (32)	10 (48)	9 (36)	

Notes. Data are n (%) unless noted. Count data are shown. Percentages are rounded to the nearest percentage point. Percentages may not add up to 100% due to rounding. Boldface indicates statistical significance (*p* < 0.05) using Fisher’s exact test. Comparisons are UC to CDS and CDS + SDMT intervention arms combined. *CDS* Clinical decision support, *SDMT* Shared decision-making tools, *UC* Usual care

^a^Breast cancer screening options included mammogram

^b^Colorectal cancer screening options included FIT, Cologuard, and colonoscopy

^c^UC compared to CDS

^d^HPV vaccination options included HPV vaccine now or later

^e^Tobacco cessation options included patch, gum, medication, cessation counselor referral, and other options

^f^UC compared to CDS + SDMT

^g^Weight management options included diabetes prevention program, nutritionist referral, medical weight mgmt. program, and other weight mgmt. program

Regarding decisions made for those respondents that had discussions (Table 4), the only significant difference between usual care and both intervention arms was that usual care respondents had significantly higher rates of reporting deciding on tobacco cessation (Freeman-Halton, *p* < 0.01). When looking at individual arm comparisons, significantly more usual care respondents reported deciding a screening option for colorectal cancer than in the CDS intervention arm (Fisher’s Exact, *p* = 0.04), and more usual care respondents reported deciding a cessation option than in the CDS + SDMT intervention arm (Fisher’s Exact, *p* = 0.02). However, no other significant differences were seen between study arms. Still, more than half of the respondents eligible for breast, colorectal, and/or tobacco cessation reported discussing those with their PCP. While 68% (223/330) of all respondents were eligible for a weight management intervention, only 32% of those reported discussing this with their PCP at the index visit. Also, only 20 (28%) of the 71 respondents eligible for cervical cancer screening reported discussing screening with their PCP. Of those 20, 16 (80%) decided to get screened (Pap smear). In contrast, of the 127 participants with a tobacco cessation recommendation, 64 (81%) reported discussing tobacco cessation at their index visit, and 34 (42%) decided on a tobacco cessation option.

### Decisional conflict and cancer prevention and screening

No significant differences were found between study arms on individual DCS items, which all showed a low level of decisional conflict (Table 5). However, 33% reported being unsure or not knowing which cancer prevention and screening options were available to them. Overall, 36% were unsure or did not know the benefits of each option available, and 48% were either unsure or did not know the risks and side effects of each option. Additionally, no significant difference was seen in mean DCS total score between usual care and combined intervention arms (*t* = 0.6, *df* = 256, *p* = 0.55) or between usual care and CDS and CDS + SDMT (*F* = 0.23, *df* = 2, *p* = 0.64) study arms.

**Table 5:** Decisional Conflict Scale

			Intervention Arms		
Survey Items	All Arms	UC	CDS	CDS + SDMT	*p*
Considering the cancer prevention and screening option(s) (breast cancer, colorectal cancer, lung cancer, cervical cancer, HPV vaccine, quitting tobacco, weight management) you discussed in your last primary care visit at Essentia, please answer the following questions:					
a. Do you know which cancer prevention and screening options are available to you?					
	(*N* = 282)	(*N* = 92)	(*N* = 100)	(*N* = 90)	0.84
Yes	189 (67)	60 (65)	74 (74)	55 (61)	
Unsure	49 (17)	16 (17)	14 (14)	19 (21)	
No	44 (16)	16 (17)	12 (12)	16 (18)	
b. Do you know the benefits of each option?					
	(*N* = 281)	(*N* = 91)	(*N* = 99)	(*N* = 91)	0.75
Yes	179 (64)	57 (63)	65 (66)	57 (63)	
Unsure	50 (18)	15 (17)	17 (17)	18 (20)	
No	52 (19)	19 (21)	17 (17)	16 (18)	
c. Do you know the risks and side effects of each option?					
	(*N* = 270)	(*N* = 88)	(*N* = 93)	(*N* = 89)	0.88
Yes	141 (52)	44 (50)	52 (56)	45 (51)	
Unsure	74 (27)	25 (29)	23 (25)	26 (29)	
No	55 (20)	19 (22)	18 (19)	18 (20)	
d. Are you clear about which benefits matter most to you?					
	(*N* = 280)	(*N* = 90)	(*N* = 99)	(*N* = 91)	0.92
Yes	173 (62)	57 (63)	63 (64)	53 (28)	
Unsure	63 (23)	19 (21)	23 (23)	21 (23)	
No	44 (16)	14 (16)	13 (13)	17 (19)	
e. Are you clear about which risks and side effects matter most to you?					
	(*N* = 277)	(*N* = 90)	(*N* = 98)	(*N* = 89)	0.69
Yes	164 (59)	50 (56)	65 (66)	49 (55)	
Unsure	68 (25)	24 (27)	21 (21)	23 (26)	
No	45 (16)	16 (18)	12 (12)	17 (19)	
f. Do you have enough support from others to make a choice?					
	(*N* = 282)	(*N* = 91)	(*N* = 99)	(*N* = 92)	0.47
Yes	228 (81)	70 (77)	82 (83)	76 (83)	
Unsure	29 (10)	12 (13)	9 (9)	8 (9)	
No	25 (9)	9 (10)	8 (8)	8 (9)	
g. Are you choosing without pressure from others?					
	(*N* = 282)	(*N* = 91)	(*N* = 99)	(*N* = 92)	0.59
Yes	233 (83)	72 (78)	81 (82)	80 (86)	
Unsure	23 (8)	9 (10)	8 (8)	6 (7)	
No	26 (9)	10 (11)	10 (10)	6 (7)	
h. Do you have enough advice to make a choice?					
	(*N* = 273)	(*N* = 89)	(*N* = 98)	(*N* = 86)	0.37
Yes	195 (71)	60 (67)	72 (74)	63 (73)	
Unsure	43 (16)	14 (16)	15 (16)	14 (16)	
No	35 (13)	15 (17)	11 (11)	9 (11)	
i. Are you clear about the best choice for you?					
	(*N* = 275)	(*N* = 89)	(*N* = 98)	(*N* = 88)	0.17
Yes	178 (65)	55 (62)	68 (69)	55 (63)	
Unsure	57 (21)	16 (18)	19 (19)	22 (25)	
No	40 (14)	18 (20)	11 (11)	11 (13)	
j. Do you feel sure about what to choose?					
	(*N* = 276)	(*N* = 89)	(*N* = 99)	(*N* = 88)	0.83
Yes	177 (64)	55 (62)	70 (71)	52 (59)	
Unsure	60 (22)	20 (23)	18 (19)	22 (25)	
No	39 (14)	14 (16)	11 (11)	14 (16)	
Total Score	(*N* = 258)	(*N* = 83)	(*N* = 90)	(*N* = 85)	
*Mean* (*95% CI*)	21.9 (18.3, 25.5)	23.1 (16.4, 29.8)	20.2 (14.4, 26.0)	22.5 (16.0, 29.0)	

Notes. Low Literacy Decisional Conflict Scale, O’Connor AM© 1993 [updated 2010]. Data are n (%) unless otherwise noted. Count data are shown. Percentages are rounded to the nearest percentage point. Percentages may not add up to 100% due to rounding. Boldface indicates statistical significance (*p* < 0.05) using chi-square test of association (df = 2). Mean total DCS scores compared using t-test/ANOVA. Comparisons are UC to CDS and CDS + SDMT intervention arms combined. *CDS* Clinical decision support, *SDMT* Shared decision making tools, *UC* Usual care

## Discussion

Our patient survey among those eligible to receive a CDS recommendation for primary and secondary cancer prevention and screening revealed few significant differences between decision aid intervention arms and the usual care arm. It is encouraging that many patients across all study arms did discuss cancer preventive care, smoking cessation, or weight management at their index visit. Our findings suggest that many patients may decide to act when PCPs discuss cancer screenings or weight management options.

Even though most participants (73%) reported having enough time to discuss cancer preventive care, many prior studies, including some of our own [19], have found that PCPs feel they often do not have enough time to discuss cancer prevention services at primary care encounters [33]. This is a notable contrast to patients in the survey, who reported they generally had enough time to discuss cancer prevention. This asymmetrical perception of time needed to discuss cancer preventive care is of interest and may deserve further exploration. Patients overwhelmingly (89%) reported their PCPs explained the benefits of the cancer prevention choices either very or somewhat well. Yet, many may not have made a choice to take part in or schedule screening the day of their visit, suggested by the low percentage (28%) of women eligible for cervical cancer screening who reported having a Pap smear done during their visit. This may also be because they prefer to receive their Pap from an OB/GYN or as part of a physical.

We discerned no consistent positive impact of either decision aid intervention (CDS or CDS + SDMT) on the provision of cancer preventive care as reported by respondents to this survey compared to usual care. In two cases usual care outperformed the CDS or CDS + SDMT. This suggests that neither CDS nor linked SDMT improved patient-reported patient perceptions of the quality of cancer prevention services in these clinical encounters. Analysis of delivery rates of preventive services is still underway, but these survey data may suggest the need to review current approaches to CDS and especially to SDMT, to assure that they are used in practice and accomplish their intended purpose effectively.

### Limitations

Our study was limited in that survey respondents reflected the predominantly White patient population served by the healthcare system. More research is needed on CDS for cancer prevention and screening with patients from other racial and ethnic groups. The survey also asked patient respondents to recall a prior primary care clinic visit. Consequently, responses may be impacted by nonresponse error, and social desirability and recall biases. We tried to mitigate these biases by surveying close to the appointment date and using unbiased language and nonleading questions. However, we were unable to assess significant differences between respondents and non-respondents because demographic survey data was unavailable from non-responders. Examining EHR data was beyond the scope of this paper. We were also not able to determine whether survey respondents from intervention clinics actually received and were exposed to study materials from either the CDS or SDMT components of the intervention. That is, the overall study was a pragmatic study, and not all PCPs or rooming staff followed the study protocol; thus, we are unable to know for certain whether patients received study materials. Rates of printing the CDS varied widely between PCPs and clinics during the study, and these rates were low (average print rate during survey timeframe = 53.75%) at the start of the intervention period. This is in part because: (a) over 50% of all patients with adult care visits were not up to date on one or more of the targeted cancer preventive services, slowing down clinic workflow; (b) clinic staff objected to initial SDMT formats, which when printed required many printed pages; and (c) various printing errors were encountered and had to be troubleshot by the study team as they arose [26]. In response, the SDMT was abbreviated and the initial and abbreviated SDMT formats are included in the Supplemental materials of this report [26]. Patients received abbreviated versions of the SDMT during the time of this survey. Due to the small number of respondents who completed the survey over the phone (*n* = 14) compared to by mail (*n* = 373), no substantive differences were able to be drawn between these two groups. We also did not account for multiple testing and perform unadjusted, simple analyses. Because this was a paper reporting exploratory patient survey results, we did not report model-based results that controlled for any potential clinic-level differences. All clinics were balanced prior to randomization [28].

## Conclusions

Compared to usual care, the CDS decision aid intervention, with or without SDMT, was not associated with significant differences in perceptions of personalized cancer prevention and screening recommendations in survey respondents. Future research will assess effects of both interventions on cancer prevention and screening as documented in the EHR. However, our findings suggest that when PCPs discuss smoking cessation or weight management options, many patients take the advice seriously and may decide to act.

## Supplementary Information

**Additional file 1.**

## Data Availability

The datasets generated and/or analysed during the current study are not publicly available due patient privacy but are available in deidentified form from the corresponding author on reasonable request.

## Abbreviations

CDS: Clinical decision support
DCS: Decisional Conflict Scale
EHR: Electronic health record
HPV: Human papillomavirus
LDCT: Low-dose computed tomography
PCP: Primary care provider
RCT: Randomized control trial
SDMT: Shared decision-making tools
