# Supplementary material for: The impact of personalized clinical decision support on primary care patients’ views of cancer prevention and screening: a cross-sectional survey
Source: BMC Health Serv Res. 2021 Jun 21;21:592. doi: 10.1186/s12913-021-06551-9 (PMC8215810; doi:10.1186/s12913-021-06551-9)
Supplement: Supplementary file 1 — Additional file 1. [file 12913_2021_6551_MOESM1_ESM.pdf]

Essentia Health
Here with you

Cancer Prevention Study at Essentia Health

Thank you for taking the time to answer the following questions. Please answer as honestly as you can. Depending on your responses, you are sometimes told to skip over some questions in this survey. When this happens you will see an arrow with a note that tells you what question to answer next.

1. In general, would you say your health is:

- ◯ Excellent
- ◯ Very good
- ◯ Good
- ◯ Fair
- ◯ Poor

2. How often do you get the social and emotional support you need?

- ◯ Always
- ◯ Usually
- ◯ Sometimes
- ◯ Rarely
- ◯ Never

3. In general, how satisfied are you with your life?

- ◯ Very satisfied
- ◯ Satisfied
- ◯ Dissatisfied
- ◯ Very dissatisfied

We would like to learn about the type of care that you received from your healthcare team at your last Essentia Health primary care appointment. Your healthcare team might include your doctors, nurses, clinical pharmacists or other staff involved in planning or delivering your care.

4. At your last primary care appointment at Essentia Health...

a. How often did your provider explain things in a way that was easy to understand?

- ◯ Always
- ◯ Usually
- ◯ Sometimes
- ◯ Rarely
- ◯ Never

b. How often did the care team talk with you about specific things you could do to prevent illness?

- ◯ Always
- ◯ Usually
- ◯ Sometimes
- ◯ Rarely
- ◯ Never

For the following questions, please think back to your last primary care visit at Essentia Health.

5. At your last primary care visit at Essentia Health, did you discuss breast cancer prevention or screening?

- ◯ Yes
- ◯ No
- ◯ Not applicable

No / Not applicable → Go to question 6 on page 3

5a. What breast cancer prevention or screening options were discussed with you? Check all that apply.

- ☐ Mammogram
- ☐ Chemotherapy
- ☐ Referral to oncology
- ☐ Referral to genetic counselors
- ☐ Doing nothing

5b. Which breast cancer prevention or screening did you do? Check all that apply.

- ☐ Mammogram
- ☐ Chemotherapy
- ☐ Referral to oncology
- ☐ Referral to genetic counselors
- ☐ Still considering options/need to learn more
- ☐ Declined breast cancer prevention or screening

Mammogram – Still considering options/need to learn more → Go to question 6 on page 3

5c. What were the reasons you did not choose a breast cancer prevention or screening option? Check all that apply.

- ☐ Side effects
- ☐ Cost
- ☐ Travel
- ☐ Not enough information
- ☐ Did not see a benefit
- ☐ Was not important to me
- ☐ I will address this at a later time
- ☐ Another reason

Please describe: ______________________________

6. At your last primary care visit at Essentia Health, did you discuss colorectal cancer prevention or screening?

- ◯ Yes
- ◯ No
- ◯ Not applicable

(No / Not applicable) → Go to question 7 on page 4

6a. What colorectal cancer prevention or screening options were discussed with you? Check all that apply.

- ☐ Stool-based fecal immunochemical test (FIT)
- ☐ Stool-based FIT DNA test (Cologuard)
- ☐ Colonoscopy
- ☐ Doing nothing

6b. Which colorectal cancer prevention or screening options did you do? Check all that apply.

- ☐ Stool-based fecal immunochemical test (FIT)
- ☐ Stool-based FIT DNA test (Cologuard)
- ☐ Colonoscopy
- ☐ Still considering options/need to learn more
- ☐ Declined colorectal cancer prevention or screening

(First four options) → Go to question 7 on page 4

6c. What were the reasons you did not choose a colorectal cancer prevention or screening option? Check all that apply.

- ☐ Side effects
- ☐ Cost
- ☐ Travel
- ☐ Not enough information
- ☐ Did not see a benefit
- ☐ Was not important to me
- ☐ I will address this at a later time
- ☐ Another reason

Please describe: ____________________

7. At your last primary care visit at Essentia Health, did you discuss <u>lung cancer prevention or screening</u>?

- ◯ Yes
- ◯ No
- ◯ Not applicable

No / Not applicable → Go to question 8 on page 5

7a. What <u>lung cancer prevention or screening</u> options were discussed with you? Check all that apply.

- ☐ Low dose CT chest scan
- ☐ Doing nothing

7b. Which <u>lung cancer prevention or screening</u> option did you do?

- ◯ Low dose CT chest scan
- ◯ Still considering options/need to learn more
- ◯ Declined lung cancer prevention or screening

Low dose CT chest scan / Still considering options → Go to question 8 on page 5

7c. What were the reasons you did not choose a <u>lung cancer prevention or screening</u> option? Check all that apply.

- ☐ Side effects
- ☐ Cost
- ☐ Travel
- ☐ Not enough information
- ☐ Did not see a benefit
- ☐ Was not important to me
- ☐ I will address this at a later time
- ☐ Another reason

Please describe: ______________________________

____

8. At your last primary care visit at Essentia Health, did you discuss cervical cancer prevention or screening?

◯ Yes
◯ No
◯ Not applicable

No / Not applicable → Go to question 9 on page 6

8a. What cervical cancer prevention or screening options were discussed with you? Check all that apply.

☐ Pap smear

☐ Doing nothing

8b. Which cervical cancer prevention or screening option did you do?

◯ Pap smear
◯ Still considering options/need to learn more
◯ Declined cervical cancer prevention or screening

Pap smear / Still considering options → Go to question 9 on page 6

8c. What were the reasons you did not choose a cervical cancer prevention or screening option? Check all that apply.

☐ Side effects
☐ Cost
☐ Travel
☐ Not enough information
☐ Did not see a benefit
☐ Was not important to me
☐ I will address this at a later time
☐ Another reason

Please describe:

____

____

3587368066

9. At your last primary care visit at Essentia Health, did you discuss Human Papilloma Virus (HPV) vaccination? (Males and females ages 18 to 26 only)

- ◯ Yes
- ◯ No
- ◯ Not applicable

(No / Not applicable) → Go to question 10 on page 7

9a. What HPV vaccination options were discussed with you? Check all that apply.

- ☐ Getting an HPV vaccination during visit
- ☐ Getting an HPV vaccination later
- ☐ Doing nothing

9b. Which HPV vaccination option did you do?

- ◯ HPV vaccination during visit
- ◯ HPV vaccination later
- ◯ Still considering options/need to learn more
- ◯ Declined HPV vaccination

(HPV vaccination during visit / HPV vaccination later / Still considering options/need to learn more) → Go to question 10 on page 7

9c. What were the reasons you did not choose an HPV vaccination option? Check all that apply.

- ☐ Side effects
- ☐ Cost
- ☐ Travel
- ☐ Not enough information
- ☐ Did not see a benefit
- ☐ Was not important to me
- ☐ I will address this at a later time
- ☐ Another reason

Please describe:

____

____

10. At your last primary care visit at Essentia Health, did you discuss tobacco cessation (quitting tobacco)?

- ◯ Yes
- ◯ No
- ◯ Not applicable

No / Not applicable → Go to question 11 on page 8

10a. What tobacco cessation options were discussed with you? Check all that apply.

- ☐ Patch
- ☐ Gum
- ☐ Medication
- ☐ Tobacco cessation counselor referral
- ☐ Other tobacco cessation options
- ☐ Doing nothing

10b. Which tobacco cessation options did you do? Check all that apply.

- ☐ Patch
- ☐ Gum
- ☐ Medication
- ☐ Tobacco cessation counselor referral
- ☐ Another tobacco cessation option
- ☐ Declined tobacco cessation

Patch / Gum / Medication / Tobacco cessation counselor referral / Another tobacco cessation option → Go to question 11 on page 8

10c. What were the reasons you did not choose a tobacco cessation option? Check all that apply.

- ☐ Side effects
- ☐ Cost
- ☐ Travel
- ☐ Not enough information
- ☐ Did not see a benefit
- ☐ Was not important to me
- ☐ I will address this at a later time
- ☐ Another reason

Please describe:

____

____

11. At your last primary care visit at Essentia Health, did you discuss <u>weight management</u>?

- ◯ Yes
- ◯ No → Go to question 12 below
- ◯ Not applicable → Go to question 12 below

11a. What <u>weight management</u> options were discussed with you? Check all that apply.

- ☐ Diabetes prevention program referral
- ☐ Nutritionist referral
- ☐ Medical weight management program
- ☐ Other weight management options
- ☐ Doing nothing

11b. Which <u>weight management</u> options did you do? Check all that apply.

- ☐ Diabetes prevention program referral → Go to question 12 below
- ☐ Nutritionist referral → Go to question 12 below
- ☐ Medical weight management program → Go to question 12 below
- ☐ Another weight management option → Go to question 12 below
- ☐ Declined weight management

11c. What were the reasons you did not choose a <u>weight management</u> option? Check all that apply.

- ☐ Side effects
- ☐ Cost
- ☐ Travel
- ☐ Not enough information
- ☐ Did not see a benefit
- ☐ Was not important to me
- ☐ I will address this at a later time
- ☐ Another reason → Please describe: ____________

If you answered "Yes" to <u>any questions 5-11</u>, complete the questions below. Otherwise, please skip to question 16 on page 9.

12. Did you have enough time to discuss the cancer prevention options mentioned above with your provider?

- ◯ Yes
- ◯ No

13. How well did your provider explain the risks of the choices available to you?

- ◯ Very well
- ◯ Somewhat well
- ◯ Not at all well

14. How well did your provider explain the benefits of the choices available to you?

- ◯ Very well
- ◯ Somewhat well
- ◯ Not at all well

15. Please consider the cancer prevention and screening option(s) you discussed in your last primary care visit at Essentia when answering the following questions.

	Yes	Unsure	No
a. Do you know which **cancer prevention and screening** options are available to you?	◯	◯	◯
b. Do you know the benefits of each option?	◯	◯	◯
c. Do you know the risks and side effects of each option?	◯	◯	◯
d. Are you clear about which benefits matter most to you?	◯	◯	◯
e. Are you clear about which risks and side effects matter most to you?	◯	◯	◯
f. Do you have enough support from others to make a choice?	◯	◯	◯
g. Are you choosing without pressure from others?	◯	◯	◯
h. Do you have enough advice to make a choice?	◯	◯	◯
i. Are you clear about the best choice for you?	◯	◯	◯
j. Do you feel sure about what to choose?	◯	◯	◯

16. What is your age?

- ◯ 18 to 26
- ◯ 27 to 39
- ◯ 40 to 49
- ◯ 50 to 59
- ◯ 60 to 69
- ◯ 70+

17. What is the highest grade or level of school that you have completed?

- ◯ 8th grade or less
- ◯ Some high school, but did not graduate
- ◯ High school graduate or GED
- ◯ Some college or 2-year degree
- ◯ 4-year college graduate
- ◯ More than 4-year college degree

18. Are you of Hispanic or Latino origin?

- ◯ Yes
- ◯ No

19. Which one or more of the following do you consider yourself? Please check all that apply.

- ☐ American Indian or Alaskan Native
- ☐ Asian
- ☐ Native Hawaiian or other Pacific Islander
- ☐ Black or African American
- ☐ White
- ☐ Some other race(s) → Please specify: ____________________

20. How many adults (18 years and older) live in your household? ____________

21. How many children (0-17 years) live in your household? ____________

22. Which of the following best describes your current employment status?

- ◯ Employed for wages
- ◯ Self-employed
- ◯ Out of work for more than 1 year
- ◯ Out of work for less than 1 year
- ◯ Homemaker
- ◯ Student
- ◯ Retired
- ◯ Unable to work
- ◯ On disability/leave of absence

23. What was your total income last year?

- ◯ Less than $26,000
- ◯ $26,000 - $51,999
- ◯ $52,000 - $74,999
- ◯ More than $75,000

24. Would you be willing to talk more about your experiences with cancer prevention screening at Essentia Health?

- ◯ Yes
- ◯ No

Thank you for your time. Your choice of a $10 Walmart e-gift card or a $10 Target gift card will be emailed or mailed to you within a week of receiving your completed survey.

◯ I prefer a Walmart e-gift card → My preferred email address is: ____________________

◯ I prefer a Target gift card → My preferred mailing address is:
____
____
____
